# Genotoxicity Response of Fibroblast Cells and Human Epithelial Adenocarcinoma In Vitro Model Exposed to Bare and Ozone-Treated Silica Microparticles

**DOI:** 10.3390/cells11020226

**Published:** 2022-01-11

**Authors:** Sabrina Colafarina, Piero Di Carlo, Osvaldo Zarivi, Massimo Aloisi, Alessandra Di Serafino, Eleonora Aruffo, Lorenzo Arrizza, Tania Limongi, Anna Poma

**Affiliations:** 1 Department of Life, Health and Environmental Sciences, University of L’Aquila, 67100 L’Aquila, Italy; sabrina.colafarina@univaq.it (S.C.); osvaldo.zarivi@univaq.it (O.Z.); massimo.aloisi@studenti.unite.it (M.A.); lorenzo.arrizza@univaq.it (L.A.); 2 Department of Psychological, Health and Territorial Sciences, University “G. d’Annunzio” of Chieti-Pescara, 66100 Chieti, Italy; piero.dicarlo@unich.it (P.D.C.); alessandra.diserafino@unich.it (A.D.S.); eleonora.aruffo@unich.it (E.A.); 3 Center for Advanced Studies and Technology-CAST, University “G. d’Annunzio” of Chieti-Pescara, 66100 Chieti, Italy; 4 Department of Applied Science and Technology, Politecnico di Torino, Corso Duca degli Abruzzi 24, 10129 Turin, Italy; tania.limongi@polito.it

**Keywords:** indoor air pollution (IAP), ozone, silica fine particles, PM 2.5, genotoxicity, A549, Hs27

## Abstract

Indoor air pollutants (IAP), which can pose a serious risk to human health, include biological pollutants, nitric oxide (NO), nitrogen dioxide (NO_2_), volatile organic compounds (VOC), sulfur dioxide (SO_2_), carbon monoxide (CO), carbon dioxide (CO_2_), silica, metals, radon, and particulate matter (PM). The aim of our work is to conduct a multidisciplinary study of fine silica particles (<2.5 μm) in the presence or absence of ozone (O_3_), and evaluate their potential cytotoxicity using MTS, micronucleus, and the comet test in two cell lines. We analyzed A549 (human basal alveolar epithelial cell adenocarcinoma) and Hs27 (human normal fibroblasts) exposed to dynamic conditions by an IRC simulator under ozone flow (120 ppb) and in the presence of silica particles (40 μg/h). The viability of A549 and Hs27 cells at 48 and 72 h of exposure to silica or silica/ozone decreases, except at 72 h in Hs27 treated with silica/ozone. The micronucleus and comet tests showed a significant increase in the number of micronuclei and the % of DNA in the queue, compared to the control, in both lines in all treatments, even if in different cell times/types. We found that silica alone or with more O_3_ causes more pronounced genotoxic effects in A549 tumor cells than in normal Hs27 fibroblasts.

## 1. Introduction

In recent years, nations all over the world have been working, more and more animatedly, to find a solution to an ever-greater problem that has been afflicting us for years, pollution. It affects, more and more negatively, not only climate change, but also public and individual health. There are many pollutants that are major disease factors in humans [1,2,3]. Closely related to the alarming level of air pollution, indoor air pollution (IAP) can represent a serious risk to human health, according to a recent World Health Organization (WHO) update, as it is considered to be the cause of the deaths of 3.8 million people annually [4]. Many authors have shown how both short- and long-term IAP exposure, by worsening indoor air quality (IAQ), can affect people’s health, leading to the onset of a whole series of disorders, such as lung cancer, chronic obstructive pulmonary disease (COPD), asthma, and digestive or neurodegenerative pathologies [5]. The best-known indoor air pollutants include biological pollutants, particulate matters (PM), and more than 400 chemical species, among which are ozone (O_3_), nitrogen oxides (NOx, NO+NO_2_), and volatile organic compounds (VOCs) [6].

Among them, PM, ranging in size from ultrafine (≤0.1 μm), to fine (0.1–2.5 μm), to coarse (2.5–10 μm) particles, is sometimes inhalable, impacting the functionality of different organs. In more detail, particulate toxicity occurs due to the size of the particles, and, moreover, PM 2.5 and smaller can penetrate the respiratory tract, up to the bronchi and bronchioles, causing adverse effects to the cardiovascular [7] and respiratory systems [8].

The toxicity of PM 2.5 is not only related to its dimensional range, but it is also related to various toxic chemicals, such as nitro PAHs/ketones/quinones, aliphatic/chlorinated hydrocarbons, polycyclic aromatic hydrocarbons (PAHs), sulphates, metals, and silica [9,10].

In recent years, public interest has been focused on atmospheric PM because of its impact on the economy [11], human health [12], and climate change [13]. In detail, the concentration of these particles and other pollutants in the atmosphere determines the extent of damage on human health itself [14]. The particles are deposited in the human respiratory tract, causing mechanical irritation in the lungs and the bronchial tubes [15]. It was demonstrated that ultrafine particles appear to be more harmful than coarse or fine particles. Due to them having lower solubility, they are not totally engulfed by pulmonary macrophages, so they then cross the pulmonary epithelial cells and, subsequently, access to the circulatory system [16,17].

The greatest risks of oxidative stress come from the ultrafine particles. It has been observed that large particles cause worsening of asthma, while the deposition of small particles affects edema and changes the heart rate [18].

Epidemiological studies conducted in the US and Europe have demonstrated the existence of a correlation between the long-term exposure of healthy adults to PM and the increase in respiratory and cardiovascular diseases, especially in urban areas [16,19].

Furthermore, it was found that exposure to particulate matter, be it chronic or acute, is also related to cardiopulmonary disorders and diseases, as well as the worsening of pre-existing respiratory problems [20,21].

High-purity silica, for high-technology applications, is obtained by the reaction in the flame between silicon tetrachloride and oxygen, SiCl_4_ + O_2_ →SiO_2_ + 2Cl_2_. In the free or combined state, silica is one of the most abundant constituents of the Earth’s crust; it can exist in different crystalline forms, amorphous and cryptocrystalline. Pure silica is a white crystalline powder that imparts slight acidity to water. The most common physical state of silica in nature is a crystalline solid, in minerals, such as quartz and its polymorphs, or, more rarely, amorphous as in opal; crystalline silica is the main constituent of different sedimentary rocks (e.g., sand, radiolarites, and quartz arenites). The chemical properties of silica, both amorphous and crystalline are similar since any differences in behavior are of a kinetic nature. Silica is practically insoluble in water, and it is resistant to acids, except for hydrofluoric acid, which converts it into volatile tetrafluoride, SiF4 [22].

Silicosis is a lung disease caused by the inhalation of crystals of silica, classified as carcinogenic to human beings, and it is the most common chronic occupational disease in the world [23,24,25,26]. Silicosis usually occurs after prolonged inhalation of small particles of free crystalline silica in mined metals (lead, anthracite, copper, silver, and gold), in foundries, in ceramic factories, and in the mining and quarrying of sandstone rocks and granite. It usually takes 20–30 years of exposure before the disease manifests, although developments can occur in <10 years if the dust exposure is very high, as in the construction of tunnels, in abrasive soap factories, and during blasting operations. In the period of 2001–2015, more than 47,000 patients were treated for silicosis in Italy [24,27]. Although working conditions have improved over the past decades, exposure to crystalline silica is still of interest today, since the silicosis risk is closely related to the concentration and size of silica in the materials used in the production processes. The limit value for occupational exposure to the respirable crystalline silica dust is 0.1 mg/m^3^ [28]. After inhalation, the silica particles interact with epithelial cells and macrophages. Although it is likely that lung macrophages that engulf the silica particles succumb to its toxic effects, the silica causes the activation and release of mediators from vital macrophages. These mediators include IL-1, TNF-alpha, fibronectin, lipid mediators, oxygen free radicals, and fibrogenic cytokines [29,30].

O_3_ is an allotropic form of oxygen; it is a molecule consisting of three oxygen atoms. It is mainly present in the stratosphere, where it is of fundamental importance for the development and maintenance of life on Earth. On the contrary, tropospheric ozone, mainly produced by reactions from precursor compounds (i.e., NOx and VOCs), is an important pollutant, and could adversely affect human health, ecosystems, vegetation, and the climate [31].

Case-control studies in humans indicate that the levels of ozone that can be found in many areas of the world induce functional and biochemical alterations, mostly of the respiratory tract and the cardiovascular system [32,33]. Recent epidemiological studies have confirmed that ozone is associated with acute and negative health effects, both in terms of morbidity and mortality [34].

Chronic exposure to ozone results in significant changes in the airways within the bronchioles. The reversibility of this type of injury is a point that is yet to be clarified. The epidemiological evidence of the chronic effects is less strong, mainly because of the absence of dedicated studies [35].

In this work, our aim was to verify the possible synergy in the generation of genotoxic damage following treatment with particulate material, such as crystalline silica, when carried out with or without the presence of ozone. Human epithelial adenocarcinoma cells and normal fibroblast cells were used as in vitro models of indoor exposure. To assess the genotoxic effects, the MTS test, the micronucleus test [36,37,38], and the comet assay were carried out. The comet assay (single-cell electrophoresis or SCGE) is used to test the genotoxic potential of substances and preparations in vitro, but also for in vivo genotoxicity tests and biomonitoring studies [39,40]. The comet assay, characterized by having high sensitivity, has a simple setup and is implemented relatively quickly [39,41]. The test can be applied to virtually any type of cell or tissue, and with a relatively large population of cells, it is able to identify the differences between single cells in relation to DNA damage [39]. Therefore, the comet assay proved suitable, even for the non-proliferating cells and specific tissue cells that represent the direct targets of environmental pollutants, such as cells of the oral mucosa, the nasal mucosa, inner epithelium of the lung, or bronchoalveolar ar washing [39].

The aim of the present work is to simulate, as faithfully as possible, what actually happens in humans from contact with the air and its particulate pollutants, through the use of a specially built cell culture room. Lichtveld et al. [42] proposed that there are inefficiencies in the classical methods of studies involving the interaction between particulate matter and in vitro cell cultures, as they involve the use of filters for the collection of the powders from air, and their extraction and dispersion directly in the culture, at predetermined concentrations. Lichtveld [42] suggested not using the classic filters to withhold some of the components but direct deposition of PM from Air, and then testing the results. Therefore, it was decided to build a controlled-atmosphere cell culture chamber, and to exploit an ozonator for injecting silica and synergic silica mixed with ozone directly onto the cells [43].

## 2. Materials and Methods

### 2.1. Analysis of the Silica Particles by SEM

The silica particles (Silicon (IV) oxide, 99.5%) were purchased from Alfa Aesar (Karlsruhe, Germany). For the morphological analysis and the microanalysis of silica particles, we used the SEM PHILIPS XL30/CP equipped with EDS (energy dispersive spectroscopy) Oxford Inca Energy 250. The purpose of EDS microanalysis is to provide specific information on the composition of the sample elements.

We inserted a carbon tape into the zero stage of the impactor to recover enough silica. The carbon tape was mounted onto an SEM stub and then coated with a thin gold film of 5 nanometers using the sputtering method. The SEM observations were carried out at different magnifications, and morphological analysis of the particles was performed simultaneously to obtain the EDS microanalysis of selected particles.

### 2.2. Culture Chamber with Controlled Atmosphere

**Figure 1:**
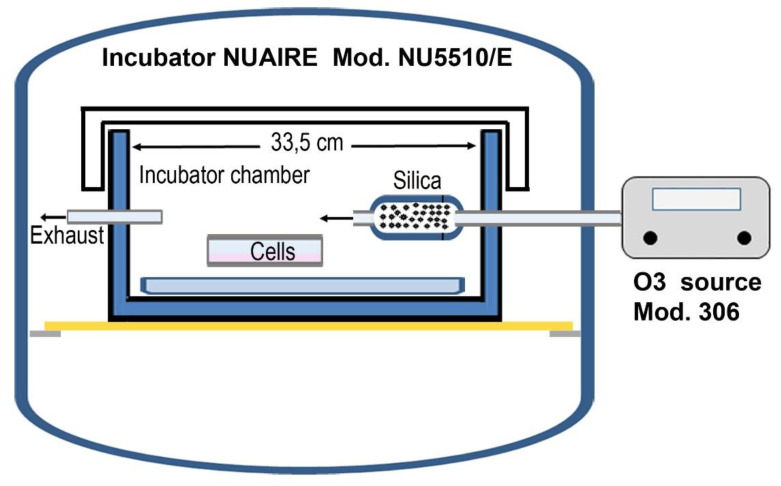
Schematic illustration of the exposure system.

The culture chamber, connected to the Ozone Calibration Source™—Model 306 (OCS™) from 2B Technologies Inc. (Boulder, CO, USA), used as a source of ozone or zero air, is placed inside an incubator NUAIRE NU-5800 Series 12 (Plymouth, MN, USA) [44]. The silica is contained in a Teflon cylinder (ø 1.8 cm × 6.7 cm) with inlet and outlet holes, with a diameter of ø 3 mm, installed inside the chamber, so as to ensure that the outlet hole is centered with respect to the chamber itself (Figure 1).

On the side walls, the following holes have been created: a ¼ hole crossed by a PFA tube of equal diameter and 65 cm length, placed between the ozonator output and the silica holder, through a flowmeter (0–2 slpm, resolution 0.1); a ⅛ hole crossed by a PFA tube with the same diameter and length of about 2 m, which acts as an overflow line for the outflow of ozone and/or silica.

During the silica/ozone treatment, the silica was blown using the ozonator flow set at an ozone concentration of 120 ppb. In the silica-only treatment, it was blown using zero air flow. The established flow is 40 µg/h.

In the case of silica, various tests were carried out in order to identify the concentration of fluxed silica, sufficient to guarantee at least 80% vitality.

### 2.3. Cells Cultures

In cell cultures treated with silica, several tests were performed to find the concentration of fluxed silica. To ensure at least 80% viability, silica was insufflated using the flow given by no air. The established flow is 40 µg/hour. In the case of silica and ozone, silica was insufflated using the flow of the ozonator set at an ozone concentration of 120 ppb.

Hs27 (human skin fibroblasts, ATCC CRL-1634) and A549 (lung alveolar adenocarcinoma epithelial, ATCC CCL-185) cell lines were purchased from the American Type Culture Collection.

The cells were seeded in monolayer in Dulbecco’s modified Eagle medium (DMEM) with 0.1 mg/mL streptomycin and 100 UI/mL penicillin, 10% bovine fetal serum and 2 mM L-glutamine (SIGMA, Milan, Italy) at a controlled atmosphere with 5% CO_2_, 90% humidity and at 37 °C. The cells were plated to the density of 2500 cells/well for MTS viability test and 10,000 cells/cm^2^ for the micronuclei and comet tests. The cells were detached with 0.05% trypsin–0.02% EDTA. All materials were purchased from SIGMA-Aldrich, Merk Life Science S.r.L., Milan, Italy.

### 2.4. Cell Viability Assay

Cell viability was determined using Cell-Titer 96^®^ Aqueous One Solution Cell Proliferation Assay (Promega) according to manufacturer’s instructions. In particular, the reaction solution contains a tetrazolium compound (3-(4,5-dimethylthiazol-2-yl)-5-(3-carboxymethoxyphenyl)-2-(4-sulfophenyl)-2H-tetrazolium, inner salt; MTS) that in the presence of an electron transporter (phenazine methosulfate; PES) is reduced by succinic dehydrogenase, to provide a soluble product in the culture medium that it absorbs at 490 nm.

Cell lines Hs27 and A549, plated 24 h earlier, were treated with crystalline silica and crystalline silica/ozone (120 ppb) at 24, 48 and 72 h.

At the time of reading, the medium (100 μL) was changed and 20 μL of Cell-Titer-Glo Reagent^®^ was added. After 20 min (time to stabilize the signal) the samples were read with a TECAN infinite F200 Elisa reader (Tecan Trading AG, Männedorf, Switzerland).

### 2.5. Cytokinesis-Block Micronucleus (CBMN) Assay

The micronuclei assay was performed according to protocol of Fenech et al. [36,37]. The cells were seeded at density of 2.5 × 10^5^. After 24 h treatments began, the cells were exposed to crystalline silica and crystalline silica/ozone (120 ppb) for 48 and 72 h, and 5 μg/mL colchicine was used as a positive control. Further, 3 μg/mL cytochalasin B was added to cells 24 h before detachment. After treatment the cells were collected, centrifuged and resuspended in PBS (1,000,000 in 200 μL), then 20 μL of suspension was distributed on SUPERFROST slides^®^ PLUS (Thermo Fisher Scientific, Braunschweig, Germany). The slides were dried and then fixed with methanol–acetic acid (3:1) for 10 min. Finally, they were stained with Giemsa 5% (SIGMA-Aldrich, Merk Life Science S.r.L., Milan, Italy) and washed in order to remove the excess dye. Once dried with xylol the slides were mounted with Balsam of Canada and observed with Leitz optical microscope magnification (400× and 1000×). One thousand binucleated cells were analyzed according to OECD guidelines [45]. For the CBMN assay three biological replicates and three technical replicates of each sample were performed.

### 2.6. Alkaline Comet Assay

Hs27 and A549 cells were seeded at a density of 2.5 × 10^5^ and, after 24 h, they were treated with crystalline silica and crystalline silica/O_3_ at 48 and 72 h. Hydrogen peroxide (100 μM) was used as a positive control, as follows: cells incubated with H_2_O_2_ for 1 h at 37 °C. After treatment the cells were collected, centrifuged, and resuspended in PBS (450,000 cells/100 μL), then 20 μL of this suspension was added to 1 mL of low-melting-point agarose (LMA), SIGMA-Aldrich, Merk Life Science S.r.L., Milan, Italy, (0.7% in PBS) and maintained at 37 °C, achieving a concentration of 90,000 cells/mL. Further, 150 μL of this suspension was distributed onto a slide previously treated with agarose 1% in PBS and left to solidify at 4 °C; this was then covered with a cover slide. Subsequently, another layer of LMA was added, covered and placed at 4 °C. After solidification the slide was placed in a lysis solution (2.5 M NaCl, 0.1 M EDTA, 1%N-lauroyl-sarcosine, 10 mM Tris-HCl pH 10, 10% DMSO, Triton 1% X-100, all reagents from SIGMA-Aldrich, Merk Life Science S.r.L., Milan, Italy). After 1 h at 4 °C, in the dark, the slides were placed in an electrophoretic chamber containing an alkaline buffer and left covered for 20 min (300 mM NaOH, 1 mM EDTA, pH > 13). In such conditions there is unfolding and denaturation of the double helix and DNA breakage at the labile sites to the alkalis to allow the DNA to unfold. Electrophoresis was performed at 20 V (theoretical current 250 mA) for 30 min in ice. At the end of the stroke the slides were washed with 0.4 M Tris-HCl buffer pH 5.0, stained with 2 μg/mL ethidium bromide (SIGMA-Aldrich, Merk Life Science S.r.L., Milan, Italy) for 5 min and then covered with a cover slide. The observations were made with a Zeiss Axioplan fluorescence microscope (Carl Zeiss. Microscopy, Jena, Germany) at 200 × magnification. Several cells from 40 to 200 were counted. For the comet assay three biological replicates and three technical replicates for each condition (control, silica, silica/ozone) were performed. The analysis of the comets was performed with CaspLab- Comet Assay Software (caslab.com, accessed on 13 December 2021). The assessment of the damage was estimated through the acquisition of various parameters, including the following: %DNA in tail and olive tail moment (OTM).

### 2.7. Statistical Analysis

To evaluate the significance of the tests, we used the t-test between control and treated for both the viability assay and for the micronucleus assay using Excel (Microsoft™ office software). 

As for the comet assay, it was decided to proceed with the help of MATLAB software (MathWorks) for running *t*-test 2 for the preparation of further graphics representative of the variability in the data.

## 3. Results

### 3.1. Silica Characterization

**Figure 2:**
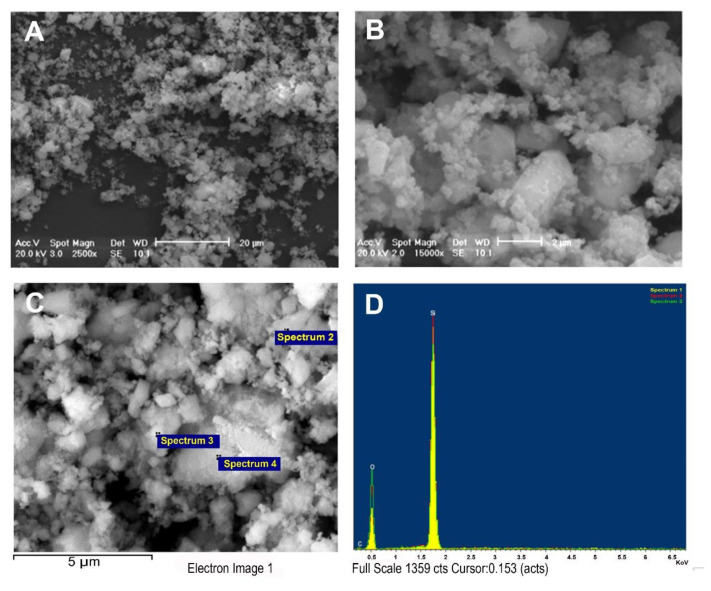
Scanning electron microscopy (SEM). Analysis of silica microparticles at different magnifications (**A**,**B**) and relative elemental spectrum with EDS (**C**,**D**).

To be able to characterize the morphology and size of the silica, a scanning electron microscope was used (SEM) (Figure 2A,B), with X-ray microanalysis (EDS) (Figure 2C,D), which allowed not only the morphological, but also the compositional information to be obtained. The powder was characterized by X-ray diffraction (XRD), as required by Italian law. The mean particle dimension was 2.5 μm.

### 3.2. MTS Test

**Figure 3:**
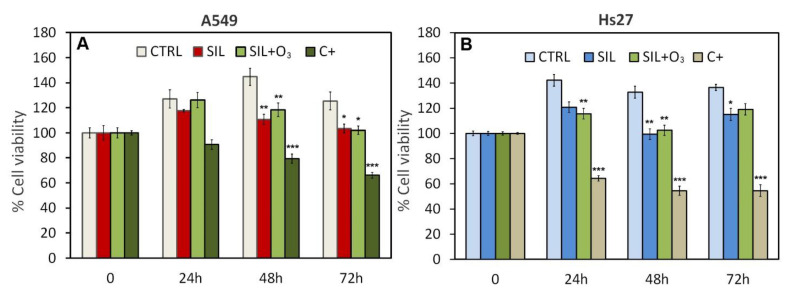
MTS assay in cell lines A549 (**A**,**B**) Hs27. Effects of silica (40 µg/h) and silica/ozone (120 ppb) on A549 and Hs27 at 24, 48, 72 h. Triton X-100 (0.1%) was used as positive control. Significance values were determined according to the Student *t*-test: * = *p* < 0.05; ** = *p* < 0.005; *** = *p* < 0.0005. All treated were compared to their respective controls. Error bars are the standard error of the mean.

In the cell line A549 at 48 h, a significant change occurred in the condition with silica and silica/ozone. In both cases, there was a reduction in the viability of about 35% and 25% compared to the control, respectively (Figure 3A). We also observed a decrease in viability of about 20% at 72 h in both treatments. At 24 h, on the contrary, there were no significant variations with any treatment. The viability in Hs27 was as follows: at 24 h, there was a decrease in viability in only the silica/ozone condition. At 48 h, however, there was a decrease of about 20% in all the conditions. At 72 h, in contrast, we found significant changes in the condition of the silica (Figure 3B).

### 3.3. Micronuclei Test

**Figure 4:**
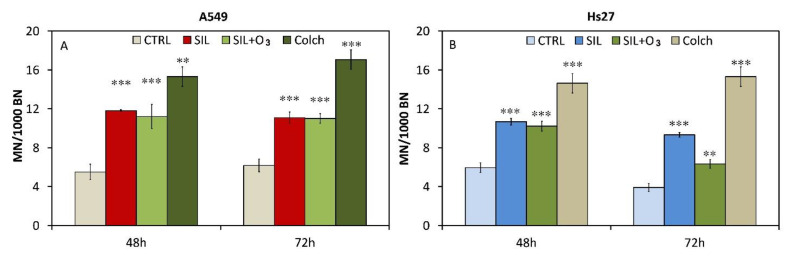
Micronuclei refers to 1000 binucleated cells (MN/1000 BN) in cell lines A549 (**A**) and HS27 (**B**): control, silica, and silica/ozone. Colchicine (0.1 μg/mL) was used as positive control. Significance values were determined according to the Student *t*-test: ** = *p* < 0.005; *** = *p* < 0.0005. All treated were compared to their respective controls. Error bars are the standard error of the mean.

The micronucleus test was performed at 48 and 72 h. Figure 4 shows the values of micronuclei in reference to 1000 binucleate cells.

It should be noted how, in both A549 and Hs27, there was a significant increase in the number of micronuclei in all the treated samples. In all the conditions, at 48 and 72 h, the number of micronuclei increased by about 100%, with respect to the controls.

### 3.4. Alkaline Comet Assay

**Figure 5:**
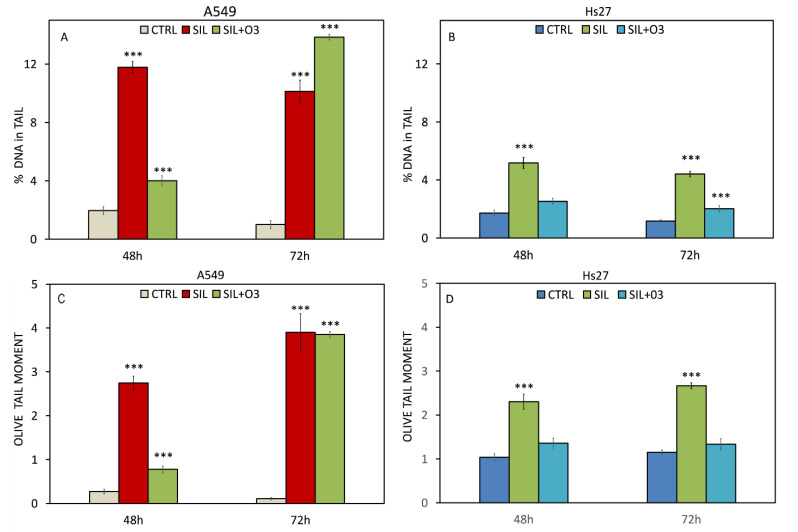
Genotoxic effect of silica and combined silica with O_3_ (120 ppb) on cell lines A549 and HS27 at 24 h and 48 h. In panel (**A**,**B**) the damage is reported, expressed as percentage DNA present in tail in A549 and HS27, respectively; in panel (**C**,**D**) olive tail moment in A549 and HS27 is reported, respectively. H_2_O_2_ 150 µM was used as positive control. Significance values were determined according to the Student *t*-test: *** = *p* < 0.0005. All treated were compared to their respective controls. Error bars are the standard error of the mean.

The comet assay was performed at 48 and 72 h. Figure 5A,B shows the % of DNA in the tail, and in C–D, the olive tail moment (OTM), for A549 and Hs27.

In all the conditions, in A549 (Figure 5A), we found significant changes in the % of DNA in the tail. In each condition, the DNA percentage in the tail (48 and 72 h) ranged from 10% to 13%, except in the silica/ozone condition at 48 h, where we observed 4% DNA in the tail.

In addition, in Hs27 (Figure 5B), there was variation in the % of DNA, except in the silica/ozone condition at 48 h. At 48 and 72 h, in the cells treated with silica, we observed about 5% DNA in the tail; at 72 h in the silica/ozone condition, the percentage of DNA in the tail was about 2.5%.

The OTM increased considerably in A549, both at 48 and 72 h, in all the treatments, indicating the presence of DNA fragmentation and, therefore, of genotoxic damage. At 48 h, it changed from 0.3 in the control to 2.8 in the treatment with silica and 0.9 with silica/ozone; at 72 h, in both treatments, the OTM was about four. In Hs27, there was only a significant difference in the cells treated with silica at both 48 and 72 h, where the OTM was greater than two in both conditions.

## 4. Discussion and Conclusions

Particulate matter has been classified as one of the main air pollutants for many years, both for dimensional variety and the variety of sources of emissions, whether natural or man-made [16,17]. In this work, we consider crystalline silica as a powder/particulate resulting from natural causes, such as erosion of rocks, and from anthropogenic sources, such as industrial activities. Furthermore, since the size of such a compound falls under the category of PM 2.5, a further hazard is introduced, given the fact that it can penetrate the tracheobronchial tract and determine the onset of silicosis [45]. Moreover, because of several epidemiological studies, crystalline silica has been classified by the IARC as carcinogenic to humans [24].

The results showed significant cytotoxic damage at 48 and 72 h, in both A549 and Hs27. These results are also confirmed at the genotoxic level; in fact, both the micronucleus test and the comet assay are extremely significant in both cell lines, at 48–72 h. It can be said that the Hs27 cells are more resistant to crystalline silica, with respect to the A549 cells; this result is not surprising, since it is known that the effects of silica are greater when there is already an inflammatory or tumoral process in progress, as in the case of lung adenocarcinoma [46]. Moreover, the clastogenic/genotoxic effect of silica/silica plus ozone on A549 could be related to a possible enhancement of the tumorigenicity of A549 and the increased levels of genomic instability. The micronucleus and comet test data indicate higher genotoxicity and clastogenicity in both A549 and Hs27 in the condition with silica alone, rather than in the silica/ozone condition. The most plausible explanation for this could be the formation of a less genotoxic silica compound in the presence of ozone and/or ozone decomposition on glass and silica surfaces, as reported in [47] and studied by means of non-porous particles on a fixed bed of silica; the ozone decomposition rate per unit of surface area was identified.

The cellular and molecular mechanism of toxicity induced by silica nanoparticles is not well characterized. Recently [48], a study elucidated the effects of silica nanoparticle exposure in three types of cells, including human aortic endothelial cells, mouse-derived macrophages, and A549 cells, using toxicogenomic analysis. Among all three cell types, the TNF and MAPK signaling pathways were upregulated by silica nanoparticles. This study suggests novelty in the evaluation of the effects of silica nanoparticle exposure in vivo and a mechanism of cytotoxicity at the level of signaling pathways. We can speculate that the same could be said for silica microparticles, such as those used in this work.

We conducted the experiments in a controlled atmosphere, at a predetermined silica flow of 40 µg/h, to ensure at least 80% cell viability, which is necessary for the execution of genotoxic tests, and with the relative presence or exclusion ozone at 120 ppb. The developed method and experimental system are extremely functional to evaluate the effects of a single component or the interaction of both. In the atmosphere, ozone interacts with atmospheric particulate, determining a multiplicity of effects; however, it remains difficult to attribute the synergistic effects in the experimental stage. Therefore, this system is suitable for the proper quantification, valuation and allocation of the various components involved in in vitro assays, with two controlled variables (silica and ozone).

## Data Availability

Data is contained within the article or Supplementary Materials.

## Supplementary Materials

The following are available online at https://www.mdpi.com/article/10.3390/cells11020226/s1, Table S1: DATA COMET, Table S2: DATA MNs, Table S3: DATA MTS.
