# Supplementary material for: Genotoxicity Response of Fibroblast Cells and Human Epithelial Adenocarcinoma In Vitro Model Exposed to Bare and Ozone-Treated Silica Microparticles
Source: Cells. 2022 Jan 11;11(2):226. doi: 10.3390/cells11020226 (PMC8773945; doi:10.3390/cells11020226)
Supplement: Supplementary file 1 [file cells-11-00226-s001.zip › cells-1529843-supplementary.pdf]

Table S1. DATA COMET.

48h A549 CTRL

glass1 TailMoment	OliveTailMoment	TailDNA%	HeadDNA%	glass2 TailMoment	OliveTailMoment	TailDNA%	HeadDNA%	glass3 TailMoment	OliveTailMoment	TailDNA%	HeadDNA%
0.0837939	0.240853	1.3966	98.6034	0.743206	0.952045	7.4321	92.5679	0.000386242	0.00212904	1.0129	98.9871
12.5808	5.65819	2.6848	97.3152	0.0727062	0.192574	1.4541	98.5459	0.00120732	0.00638095	2.0402	97.9598
0.687305	0.99179	0.7275	99.2725	0.0592856	0.194677	1.4821	98.5179	0.00990962	0.0460974	0.3303	99.6697
0.0311727	0.147179	1.0391	98.9609	0.0209174	0.100545	0.6972	99.3028	0.000515047	0.00281565	0.0172	99.9828
0.00836849	0.0446234	0.2789	99.7211	0.0309552	0.11505	0.7739	99.2261	0.0261524	0.11448	40.8717	59.1283
0.000162557	0.00106572	0.7054	99.2946	0.0697034	0.236078	1.7426	98.2574	0.0117561	0.0541383	1.2939	98.7061
0.0520807	0.201323	1.302	98.698	0.0423118	0.171755	1.4104	98.5896	0.000221673	0.00150823	1.0074	98.9926
0.0663491	0.210609	1.327	98.673	0.0290071	0.12874	0.9669	99.0331	0.00566827	0.0332337	20.1889	79.8111
0.00721742	0.0394402	0.2406	99.7594	0.144903	0.405981	2.4151	97.5849	0.0078585	0.0442793	3.262	96.738
0.235508	0.511873	3.3644	96.6356	0.000409308	0.00252372	0.0136	99.9864	0.0261038	0.119083	0.8701	99.1299
0.000334039	0.00218213	0.0111	99.9889	0.0064901	0.0346305	0.2163	99.7837	0.0149701		0.499	99.501
0.0327075	0.139752	1.0903	98.9097	0.000455973	0.0025487	0.0152	99.9848	0.389877	0.657676	1.8735	98.1265
0.00576509	0.0283491	0.1922	99.8078	0.000601382	0.00310061	0.02	99.98	0.0142912	0.0676163	0.4764	99.5236
0.000898431	0.00498644	0.0299	99.9701	0.000430334	0.00238988	0.0143	99.9857	0.0210104	0.0990322	1.7003	98.2997
0.000787386	0.00429075	0.0262	99.9738	0.000246079	0.00150338	0.0082	99.9918	0.0106399	0.0468863	1.3547	98.6453
0.000482911	0.00256611	0.0161	99.9839	0.0121659	0.0644387	0.4055	99.5945	0.0059585	0.0314761	0.1986	99.8014
0.000655546	0.00345107	10.0219	89.9781	0.000225539	0.00137797	0.0075	99.9925	0.743206	0.952045	0.4321	99.5679
0.0180831	0.0748289	0.6028	99.3972	4.84595	3.45018	17.948	82.052	0.0727062	0.192574	1.4541	98.5459
0.0455937	0.173469	1.5198	98.4802	0.000277025	0.00174336	0.0092	99.9908	0.0592856	0.194677	1.4821	98.5179
0.000523048	0.00292159	1.0174	98.9826	0.0435848	0.160337	0.8717	99.1283	0.0209174	0.100545	0.6972	99.3028
0.059493	0.207962	1.9831	98.0169	0.763441	1.08149	6.9404	93.0596	0.0309552	0.11505	0.7739	99.2261
0.0334176	0.132881	1.1139	98.8861	0.000297365	0.00184861	0.0099	99.9901	0.0697034	0.236078	1.7426	98.2574
0.190633	0.474977	2.7233	97.2767	0.0132039	0.0722337	0.4401	99.5599	0.0423118	0.171755	1.34104	98.65896
0.0148474	0.0697285	10.4949	89.5051	0.0284647	0.145948	0.9488	99.0512	0.0290071	0.12874	1.9669	98.0331
0.0331058	0.144651	3.1035	96.8965	0.000420388	0.00255131	0.014	99.986	0.144903	0.405981	1.4151	98.5849
0.011108	0.0527376	3.3703	96.6297	0.000175812	0.00107831	0.0059	99.9941	0.000409308	0.00252372	8.0136	91.9864
0.0328579	0.154789	1.0953	98.9047	8.86E-05	0.000606814	0.003	99.997	0.0064901	0.0346305	0.2163	99.7837
0.0117136	0.0547576	0.3905	99.6095	0.000252423	0.00156841	0.0084	99.9916	0.000455973	0.0025487	0.0152	99.9848
0.0960175	0.309238	0.4004	99.5996	0.000297692	0.00197166	0.0099	99.9901	0.000601382	0.00310061	4.02	95.98
0.020438	0.0906069	0.6813	99.3187	0.0109125	0.05543	0.3637	99.6363	0.000430334	0.00238988	1.0143	98.9857
0.0243804	0.111884	0.8127	99.1873	0.000321231	0.0019899	0.0107	99.9893	0.000246079	0.00150338	0.0082	99.9918
0.0370148	0.125807	0.9254	99.0746	0.000156772	0.000967392	0.0052	99.9948	0.0121659	0.0644387	0.4055	99.5945
0.0192964	0.0896772	0.6432	99.3568	0.0837939	0.240853	1.3966	98.6034	0.000225539	0.00137797	3.0075	96.9925
0.399004	0.684669	1.9876	98.0124	12.5808	5.65819	30.6848	69.3152	4.84595	3.45018	2.948	97.052
0.007636	0.0373479	5.2545	94.7455	0.687305	0.99179	5.7275	94.2725	0.000277025	0.00174336	0.0092	99.9908
0.000433409	0.00248463	1.0144	98.9856	0.0311727	0.147179	1.0391	98.9609	0.0435848	0.160337	0.8717	99.1283
0.765876	1.09327	1.9625	98.0375	0.00836849	0.0446234	0.2789	99.7211	0.763441	1.08149	1.9404	98.0596
0.000525719	0.00281632	2.0175	97.9825	0.000162557	0.00106572	0.0054	99.9946	0.000297365	0.00184861	1.0099	98.9901
0.000502993	0.00279031	7.0168	92.9832	0.0520807	0.201323	1.302	98.698	0.0132039	0.0722337	0.4401	99.5599
0.000263578	0.00151227	3.0088	96.9912	0.0663491	0.210609	1.327	98.673	0.0284647	0.145948	0.9488	99.0512
0.0282635	0.119668	0.9454	99.0546	0.00721742	0.0394402	0.2406	99.7594	0.000420388	0.00255131	0.014	99.986
0.000269122	0.0016505	1.009	98.991	0.235508	0.511873	3.3644	96.6356	0.000175812	0.00107831	2.0059	97.9941
0.000585261	0.00323443	1.0195	98.9805	0.000334039	0.00218213	0.0111	99.9889	8.86E-05	0.000606814	1.003	98.997
0.000505946	0.00280643	2.0169	97.9831	0.0327075	0.139752	1.0903	98.9097	0.000252423	0.00156841	0.0084	99.9916
0.00617392	0.0298151	1.2058	98.7942	0.00576509	0.0283491	0.1922	99.8078	0.000297692	0.00197166	0.0099	99.9901
0.00581917	0.0292999	0.194	99.806	0.000898431	0.00498644	0.0299	99.9701	0.0109125	0.05543	0.3637	99.6363
0.00144134	0.00787025	5.048	94.952	0.000787386	0.00429075	0.0262	99.9738	0.000321231	0.0019899	0.0107	99.9893
0.0546656	0.193423	1.3666	98.6334	0.000482911	0.00256611	0.0161	99.9839	0.000156772	0.000967392	0.0052	99.9948
0.00745399	0.0348226	1.2485	98.7515	0.000655546	0.00345107	0.0219	99.9781	0.0147269	0.0797288	0.4909	99.5091
0.000425644	0.00249438	1.0142	98.9858	0.0180831	0.0748289	0.6028	99.3972	0.0239963	0.109116	2.7999	97.2001
0.0100674	0.0571497	0.3456	99.6544	0.0455937	0.173469	1.5198	98.4802	0.000667959	0.0034888	0.0223	99.9777
0.00046812	0.00265431	2.0156	97.9844	0.000523048	0.00292159	0.0174	99.9826	0.0547935	0.174424	1.0959	98.9041
0.00005628	0.00184507	0.0119	99.9881	0.059493	0.207962	1.9831	98.0169	0.00889676	0.0451042	0.2966	99.7034
0.0410741	0.159584	1.3691	98.6309	0.0334176	0.132881	1.1139	98.8861	0.00632385	0.0413932	0.2108	99.7892
0.0156543	0.074375	3.5218	96.4782	0.190633	0.474977	2.7233	97.2767	0.0426623	0.190473	1.0666	98.9334
0.0174457	0.0876735	0.5815	99.4185	0.0148474	0.0697285	0.4949	99.5051	0.0103216	0.061262	0.3441	99.6559
0.000474809	0.00264337	0.0158	99.9842	0.0331058	0.144651	1.1035	98.8965	0.000261879	0.00150693	1.0087	98.9913
0.000409198	0.00231708	1.0136	98.9864	0.011108	0.0527376	0.3703	99.6297	0.000421182	0.00271273	1.014	98.986
0.032299	0.15264	1.0766	98.9234	0.0328579	0.154789	1.0953	98.9047	0.0205597	0.111187	0.6853	99.3147
0.0932896	0.241718	1.5548	98.4452	0.0117136	0.0547576	0.3905	99.6095	0.00446379	0.0292979	4.1488	95.8512
0.0118395	0.055936	16.3947	83.6053	0.0960175	0.309238	2.4004	97.5996	0.0460603	0.218546	1.5353	98.4647
0.000238097	0.00138186	0.0079	99.9921	0.020438	0.0906069	0.6813	99.3187	0.00026737	0.00173257	10.0089	89.9911
0.00115522	0.00625442	0.0385	99.9615	0.0243804	0.111884	0.8127	99.1873	0.000236369	0.00162792	2.0079	97.9921
0.00542064	0.0278875	0.1807	99.8193	0.0370148	0.125807	0.9254	99.0746	0.000452095	0.00289789	2.0151	97.9849
0.0141872	0.0702666	0.4729	99.5271	0.0192964	0.0896772	0.6432	99.3568	0.000529538	0.00297	1.0177	98.9823
0.000386242	0.00212904	1.0129	98.9871	0.399004	0.684669	4.9876	95.0124	0.0163317	0.070199	5.5444	94.4556
0.00120732	0.00638095	2.0402	97.9598	0.007636	0.0373479	0.2545	99.7455	0.0273574	0.121188	1.9119	98.0881
0.00990962	0.0460974	0.3303	99.6697	0.000433409	0.00248463	0.0144	99.9856	0.0375805	0.153607	2.2527	97.7473
0.000515047	0.00281565	1.0172	98.9828	0.765876	1.09327	6.9625	93.0375	0.000687325	0.0035185	2.0229	97.9771
0.0261524	0.11448	0.8717	99.1283	0.000525719	0.00281632	0.0175	99.9825	0.351871	0.723221	0.9097	99.0903
0.0117561	0.0541383	0.2939	99.7061	0.000502993	0.00279031	0.0168	99.9832	7.88187	8.20828	28.1495	71.8505

N=259

	TailMoment
MEAN	0.259719076
SD	1.309693141
SE	0.081380372

	OliveTailMoment
MEAN	0.271996093
SD	0.853209998
SE	0.05301589

	TailDNA%
MEAN	1.96443722
SD	4.336454769
SE	0.269454189

	HeadDNA%
MEAN	98.03556278
SD	4.344850623
SE	0.269975882

T-test OTM	
Ctrl 48h vs SIL 48h	1.20E-18
ctrl 48h vs SI-O348h	2.61864E-05

T-test TailDNA%	
Ctrl 48h vs SIL 48h	2.24E-48
ctrl 48h vs SI-O348h	0.463281283

OTM T-TEST

ctrl 48h	silica 48h	silica-O3
0.00212904	0.95205	0.00165268
0.00638095	0.19257	0.264183
0.0460974	0.19468	0.212384
0.00281565	0.10055	0.316402
0.11448	0.11505	0.0029606
0.0541383	0	0.00333191
0.00150823	0.17176	0.00134621
0.0332337	0.12874	0.484449
0.0442793	0.40598	0.125046
0.119083	0.00252	0.00255877
	0.03463	0.171477
0.657676	0.00255	0.501348
0.0676163	0.0031	0.00277325
0.0990322	0.00239	0.245235
0.0468863	0.0015	0.00264807
0.0314761	0.06444	0.131855
0.952045	0.00138	0.132943
0.192574	3.45018	0.104006
0.194677	0.00174	0.084715
0.100545	0.16034	0.189494
0.11505	1.08149	0.00251616
0.236078	0.00185	1.05558
0.171755	0.07223	0.137339
0.12874	0.14595	0.413271
0.405981	0.00255	0.55145
0.00252372	0.00108	0.00317879
0.0346305	0.00061	0.00292391
0.0025487	0.00157	0.126128
0.00310061	0.00197	0.00196338
0.00238988	0.05543	0.267601
0.00150338	0.00199	0.00139014
0.0644387	0.00097	0.0563727
0.00137797	0.24085	0.12718
3.45018	5.65819	0.00327817
0.00174336	0.99179	0.726817
0.160337	0.14718	0.00218839
1.08149	0.04462	0.024306
0.00184861	0.00107	0.208663
0.0722337	0.20132	1.72951
0.145948	0.21061	0.624216
0.00255131	0.03944	0.231295
0.00107831	0.51187	0.276852
0.000606814	0.00218	0.00191539
0.00156841	0.13975	0.213553
0.00197166	0.02835	0.00356723
0.05543	0.00499	0.00344538
0.0019899	0.00429	0.37213
0.000967392	0.00257	0.00256618
0.0797288	0.00345	0.201788
0.109116	0.07483	0.00318445
0.0034888	0.17347	0.0788441
0.174424	0.00292	0.00215227
0.0451042	0.20796	0.00283794
0.0413932	0.13288	0.0015848
0.190473	0.47498	0.108236
0.061262	0.06973	0.273885
0.00150693	0.14465	0.00324875
0.00271273	0.05274	0.0474304
0.111187	0.15479	0.0772751
0.0292979	0.05476	0.553568
0.218546	0.30924	0.00211381
0.00173257	0.09061	0.0567873
0.00162792	0.11188	0.0570826
0.00289789	0.12581	0.0790248
0.00297	0.08968	0.0893492
0.070199	0.68467	0.601431
0.121188	0.03735	0.349138
0.153607	0.00248	0.150033
0.0035185	1.09327	0.072109

TailDNA% T-TEST

ctrl 48h	silica 48h	silica-O3
1.3274	12.7609	0.753334
2.68653	23.6682	0.00840987
0.231735	13.1427	2.3326
0.498249	21.4864	1.67068
0.0176447	19.2183	2.86211
0.639947	16.5442	0.0191174
0.323925	35.2711	0.0199808
0.334022	13.0926	0.00880709
2.04021	8.36157	3.32777
0.347051	4.52661	119.314
0.016851	16.1308	0.0163639
0.880609	17.7244	1.59927
0.244593	16.7944	3.88373
0.0150034	11.7957	0.0169343
0.0216968	9.02528	1.76458
1.26284	3.19287	0.0169995
11.1107	6.6567	0.944388
0.0141414	5.16432	0.867485
0.902395	4.85455	0.577675
1.38498	1.52322	0.67903
0.766128	12.0219	1.67377
1.12818	2.58193	0.0157282
0.186273	7.85231	8.48743
10.6492	10.9497	1.18266
2.32053	18.3007	2.7739
3.11777	9.81078	5.00234
0.0246881	9.75615	0.0207508
0.0152287	11.6123	0.016834
0.277741	11.0946	0.924669
0.23562	11.3623	0.0116826
0.488692	9.20988	2.30392
0.0174412	12.5486	0.00844207
0.0209256	9.14417	0.425194
1.19318	6.0484	0.96511
5.75632	6.47041	0.021136
1.48881	9.39596	4.67557
0.839773	9.38934	0.0122339
2.30029	4.68133	0.173509
7.84942	2.14502	1.28873
3.58262	15.4723	19.1763
0.575788	24.1085	3.96296
1.27893	27.3688	2.16187
1.24968	15.2393	2.51326
1.93507	9.22208	0.010499
1.1081	16.1047	1.52965
0.883299	7.07587	0.0226926
5.93156	9.65089	0.0235613
0.920207	7.82283	3.48765
1.07612	9.77872	0.0168304
1.05054	13.575	1.59699
0.798912	18.43	0.0191559
1.45939	16.3072	0.630501
0.0219545	32.9217	0.0128138
0.605871	13.1105	0.0192889
3.32425	15.8508	0.0102486
0.175443	14.7128	0.777148
0.0153264	16.6406	2.43142
1.38196	10.8889	0.0210802
0.895985	6.51154	0.322663
0.388385	9.09412	0.585488
16.6532	7.80812	4.36849
0.0117798	4.63668	0.0118155
0.193342	6.13187	0.431366
0.77987	4.55114	0.426115
0.519205	2.58248	0.594751
1.61276	11.2484	0.509993
1.13456	5.0735	4.5774
0.7866	23.9344	2.88292
1.14618	5.76535	1.62532

Table S2: DATA MNs.

HS27 ctrl	48h		72h	
	binucleated	micronuclei	binucleated	micronuclei
glass 1	995	6	1775	6
glass 2	823	4	1183	4
glass 3	1022	7	983	5
glass 4	1314	9	1367	7
glass 5	932	4	891	3
glass 6	1031	7	1266	4

HS27 Silica	48h		72h	
	binucleated	micronuclei	binucleated	micronuclei
glass 1	419	4	228	2
glass 2	528	6	504	5
glass 3	612	7	693	6
glass 4	812	9	781	7
glass 5	762	8	734	7
glass 6	692	7	811	8

HS27 Silica + O_3	48h		72h	
	binucleated	micronuclei	binucleated	micronuclei
glass 1	587	6	654	5
glass 2	621	7	765	4
glass 3	437	4	542	3
glass 4	768	9	679	5
glass 5	676	6	701	4
glass 6	598	6	769	5

HS27 ctrl	48h		72h	
	binucleated	micronuclei	binucleated	micronuclei
glass 1	872	12	1475	24
glass 2	963	15	1283	21
glass 3	1128	18	1421	19
glass 4	1423	21	1329	19
glass 5	769	11	1091	17
glass 6	1192	16	1127	18

HS27 ctrl	micronuclei/1000 binucleated cells	
	48h	72h
glass 1	6.0301508	3.3802817
glass 2	4.8602673	3.3812342
glass 3	6.8493151	5.08647
glass 4	6.8493151	5.1207023
glass 5	4.2918455	3.3670034
glass 6	6.7895247	3.1595577
MEAN	5.9450697	3.9158749
SD	1.1194292	0.9238916
SE	0.500624	0.4131769

HS27 Silica	micronuclei/1000 binucleated cells	
	48h	72h
glass 1	9.5465394	8.7719298
glass 2	11.363636	9.9206349
glass 3	11.437908	8.6580087
glass 4	11.083744	9.1984231
glass 5	10.498688	9.5367847
glass 6	10.115607	9.864365
MEAN	10.674354	9.3250244
SD	0.753757	0.5398767
SE	0.3370904	0.2414402

HS27 Silica + O_3	micronuclei/1000 binucleated cells	
	48h	72h
glass 1	10.22146508	7.6452599
glass 2	11.27214171	5.2287582
glass 3	9.153318078	5.5350554
glass 4	11.71875	7.3637703
glass 5	8.875739645	5.7061341
glass 6	10.03344482	6.5019506
MEAN	10.21247655	6.3301547
SD	1.125394627	1.0061844
SE	0.503291777	0.4499794

HS27 ctrl	micronuclei/1000 binucleated cells	
	48h	72h
glass 1	13.761468	16.271186
glass 2	15.576324	16.367888
glass 3	15.957447	13.370866
glass 4	14.757554	14.296464
glass 5	14.304291	15.582035
glass 6	13.422819	15.971606
MEAN	14.629984	15.310007
SD	0.9991631	1.2117945
SE	0.4468393	0.541931

HS27	48h	72h
CTRL	5.9450697	3.9158749
SIL	10.674354	9.3250244
SIL+O3	10.212477	6.3301547
Colch	14.629984	15.310007
SE	0.500624	0.4131769
SE	0.3370904	0.2414402
SE	0.5032918	0.4499794
SE	0.4468393	0.541931

Ctrl Hs27 vs Trt Hs27

	Ctrl	Silica	Silica+ O3	Colch	Ctrl	Silica	Silica+ O3	Colch
	48h	48h	48h	48h	72h	72h	72h	72h
	6.030150754	9.5465394	10.221465	13.76146789	3.3802817	8.7719298	7.645259939	16.27118644
	4.860267315	11.363636	11.272142	15.57632399	3.3812342	9.9206349	5.22875817	16.36788776
	6.849315068	11.437908	9.1533181	15.95744681	5.08647	8.6580087	5.535055351	13.37086559
	6.849315068	11.083744	11.71875	14.75755446	5.1207023	9.1984231	7.36377025	14.29646351
	4.291845494	10.498688	8.8757396	14.30429129	3.3670034	9.5367847	5.706134094	15.58203483
	6.789524733	10.115607	10.033445	13.42281879	3.1595577	9.864365	6.501950585	15.97160603
T-Test		6.32E-06	6.192E-05	6.002486E-08		2.174E-07	0.001491383	5.06272E-09
		***	***	***		***	**	***

Table S3: DATA MTS.

MTS_HS27

CTRL	0	24h	48h	72h
	0.401199996	0.5019	0.46095	0.527535
	0.402999997	0.6527	0.516666	0.59227
	0.401800007	0.50529	0.51899	0.53208
	0.366299987	0.456177	0.65219	0.475897
	0.401199996	0.555736	0.54995	0.558635
	0.402999997	0.62253	0.573722	0.505488
	0.401800007	0.4927	0.49131	0.532769
	0.366299987	0.650889	0.418566	0.496551
	0.3577	0.543778	0.46435	0.55976
mean	0.389144442	0.5535222	0.5162993	0.5312205
SD	0.019453288	0.0727539	0.0696796	0.0357678
SE	0.006877776	0.0257224	0.0246354	0.0126458

SILICA	0	24h	48h	72h
	0.401199996	0.518999	0.447295	0.575021
	0.402999997	0.494278	0.447777	0.500822
	0.401800007	0.447459	0.389624	0.47074
	0.366299987	0.451811	0.420512	0.478933
	0.422	0.604229	0.462223	0.42512
	0.408	0.436712	0.361112	0.39082
	0.428	0.451102	0.356433	0.40931
	0.4	0.455666	0.387222	0.52166
	0.377	0.534229	0.342113	0.51877
mean	0.403787498	0.4882761	0.4015902	0.4643951
SD	0.018387145	0.0554939	0.0444709	0.0598518
SE	0.006949688	0.0209747	0.0168084	0.0226218

SILICA+O3	0	24h	48h	72h
	0.401199996	0.4394	0.357	0.5683
	0.402999997	0.5291	0.4887	0.4814
	0.401800007	0.4131	0.4273	0.5238
	0.366299987	0.45689	0.40123	0.50933
	0.401199996	0.3482	0.3234	0.4273
	0.402999997	0.454	0.3969	0.4206
	0.401800007	0.458	0.4254	0.3873
	0.366299987	0.48922	0.4105	0.4173
	0.397223	0.50243	0.398223	0.479883
mean	c	0.4544822	0.4031837	0.468357
SD	0.015533796	0.0528297	0.0461086	0.0593588
SE	0.005492026	0.0186781	0.0163018	0.0209865

C+	0	24h	48h	72h
	0.391199996	0.2114	0.1951	0.1834
	0.382999997	0.2212	0.1864	0.1648
	0.411800007	0.2058	0.1786	0.1681
	0.366299987	0.2219	0.1947	0.1947
	0.391199996	0.2157	0.1752	0.1874
	0.392999997	0.2113	0.1597	0.1936
mean	0.333785712	0.21455	0.181617	0.182
SD	0.014803846	0.006271	0.013465	0.012775
SE	0.006043645	0.00256	0.005497	0.005215

CTRL		0	24h	48h	72h
	CTRL	0.3891	0.5535	0.5163	0.5312
	SIL	0.4038	0.4883	0.4016	0.4644
	SIL+O3	0.3931	0.4545	0.4032	0.4684
	C+	0.3338	0.2146	0.1816	0.1820
	SE	0.0069	0.0257	0.0246	0.0126
	SE	0.0069	0.0210	0.0168	0.0226
	SE	0.0055	0.0187	0.0163	0.0210
	SE	0.0022	0.0076	0.0067	0.0086

NORMALIZED VALUE

	0	24h	48h	72h
CTRL	100	142.2408	132.6755	136.5099
SIL	100	120.9240	99.4558	115.0098
SIL+O	100	115.6223	102.5717	119.1521
C+	100	64.2778	54.4112	54.5260
SE	1.7674	4.6470	4.7715	2.3805
SE	1.7211	4.2957	4.1855	4.8712
SE	1.3972	4.1098	4.0433	4.4809
SE	0.6717	2.2845	3.6644	4.7075

	T-Test	
SIL 24 vsCtrl 24	0.048186492	
SIL 48 vsCtrl 24	0.000732954	**
SIL 72 vsCtrl 24	0.032475219	*
SIL+O3 24 vsCtrl 24	0.00447594	**
SIL+O3 48 vsCtrl	0.000907095	**
SIL+O3 72 vsCtrl	0.015098933	
C+ vs C 24h	4.54778E-08	***
C+ vs C 48h	3.53362E-08	***
C+ vs C 72h	7.53164E-12	***

MTS_A549

CTRL	0	24h	48h	72h
	0.93809998	1.2627	1.6001	1.437
	1.078299999	1.3286	1.6106	1.2636
	0.930499971	1.376	1.323	1.0379
	0.902499974	1.34544	1.2134	0.9453
	0.93387	0.978	1.3959	1.34601
	1.03452	1.2803	1.413	1.3456
	0.9856	1.262	0.956	1.2457
	1	0.956	1.43498	1.3798
	0.88777	1.26665	1.6433	0.91211
mean	*0.966651103*	*1.2284*	*1.3989*	*1.2126*
SD	*0.07880126*	*0.1537*	*0.2188*	*0.1968*
SE	*0.03940063*	*0.0887*	*0.0977*	*0.0868*

SILICA	0	24h	48h	72h
	0.93809998	1.208	1.104	*0.932*
	1.078299999	1.1696	1.1544	1.039
	0.930499971	1.183	1.00435	0.9383
	0.802499974	1.2878	0.959	1.016
	0.9387	1.1803	1.013	0.973
	1.03452	1.062	1.256	0.9134
	0.9856	0.956	0.952	1.1461
	1	0.954	0.961	1.1456
	0.9777	1.168	1.1746	0.8357
		1.237		
mean	*0.960279992*	*1.1299*	*1.0643*	*0.9932*
SD	*0.112692675*	*0.0195*	*0.0764*	*0.06*
SE	*0.056346338*	*0.0113*	*0.0441*	*0.0347*

SILICA+O3	0	24h	48h	72h
	0.93809998	1.1955	0.9289	1.1656
	1.078299999	1.2949	1.3572	0.8839
	0.930499971	1.4485	1.0647	0.7983
	0.992499974	1.4287	1.1402	1.2091
	1.19875	1.14534	0.9948	0.98123
	0.9743	1.19832	1.1598	0.8948
	0.9823	0.92387	1.2987	1.03872
	1.2398	0.9861	1.02398	0.89321
	1.2987	1.3211	1.29871	0.98765
mean	*0.963324992*	*1.2158*	*1.1408*	*0.9836*
SD	*0.07880126*	*0.1275*	*0.1791*	*0.2037*
SE	*0.03940063*	*0.0736*	*0.0623*	*0.0332*

C+	0	24h	48h	72h
	0.98809998	0.8221	0.7256	0.5606
	1.078299999	0.8191	0.6915	0.5588
	0.930499971	0.8032	0.7481	0.5374
	0.992499974	0.78956	0.6806	0.5656
	1.19875	0.77636	0.6671	0.617
	0.9743	0.7823	0.6882	0.6547
mean	0.880349989	0.79877	0.70018	0.58235
SD	0.096873864	0.01916	0.03046	0.04419
SE	0.039548589	0.00782	0.01244	0.01804

CTRL		0	24h	48h	72h
	CTRL	*0.9667*	*1.2284*	*1.3989*	*1.2126*
	SIL	*0.9603*	*1.1299*	*1.0643*	*0.9932*
	SIL+O3	*0.9633*	*1.2158*	*1.1408*	*0.9836*
	C+	*0.8803*	*0.7988*	*0.7002*	*0.5823*
	SE	*0.0394*	*0.0887*	*0.0977*	*0.0868*
	SE	*0.0563*	*0.0113*	*0.0441*	*0.0347*
	SE	*0.0394*	*0.0736*	*0.0623*	*0.0332*
	SE	*0.0161*	*0.0300*	*0.0254*	*0.0136*

NORMALIZED VALUE

	0	24h	48h	72h
CTRL	100	127.0789	144.7182	125.4390
SIL	100	117.6590	110.8282	103.4316
SIL+O3	100	126.2102	118.4207	102.1060
C+	100	90.7332	79.5347	66.1498
SE	4.0760	7.2219	6.9825	7.1565
SE	5.8677	0.9959	4.1425	3.4901
SE	4.0901	6.0528	5.4646	3.3776
SE	1.8271	3.7612	3.6347	2.3290

	T-Test	
SIL 24 vsCtrl 24	0.17123048	**
SIL 48 vsCtrl 24	0.0008545	**
SIL 72 vsCtrl 24	0.00936275	
SIL+O3 24 vsCtrl	0.87532283	*
SIL+O3 48 vsCtrl	0.01017177	*
SIL+O3 72 vsCtrl	0.01103546	
C+vs C 24h	1.4043E-05	***
C+vs C 48h	3.4919E-06	***
C+vs C 72h	3.7565E-06	***
